# Guided Genioplasty: Comparison between Conventional Technique and Customized Guided Surgery

**DOI:** 10.3390/jpm13121702

**Published:** 2023-12-12

**Authors:** Raúl Antúnez-Conde Hidalgo, José Luis Silva Canal, Carlos Navarro Cuéllar, Celia Sánchez Gallego-Albertos, Javier Arias Gallo, Ignacio Navarro Cuéllar, Antonio López Davis, Gastón Demaria Martínez, Néstor Naranjo Aspas, José Zamorano León, Manuel Chamorro Pons

**Affiliations:** 1 Maxillofacial Surgery Department, Hospital Universitario Ruber Juan Bravo, 28006 Madrid, Spain; raul@antunezcondemaxilofacial.com (R.A.-C.H.); joseluis_silva92@hotmail.com (J.L.S.C.); drasanchezgallego@gmail.com (C.S.G.-A.); javierariasgallo@gmail.com (J.A.G.); alpzdvs@gmail.com (A.L.D.); gastondemaria@gmail.com (G.D.M.); nestornaranjo.lp@gmail.com (N.N.A.); mchamorropons@hotmail.com (M.C.P.); 2 Maxillofacial Surgery Department, Hospital General Universitario Gregorio Marañón, 28007 Madrid, Spain; ignacio.navarro@salud.madrid.org; 3 Surgery Department, Universidad Complutense de Madrid, 28040 Madrid, Spain; 4 Department of Public Health and Maternal and Child Health, Faculty of Medicine, Universidad Complutense de Madrid, 28040 Madrid, Spain; jjzamorano@ucm.es

**Keywords:** genioplasty, chin deformity, virtual surgical planning, CAD-CAM technology, stereolithographic models, patient-specific plates, head and neck reconstruction, aesthetic surgery

## Abstract

Background: Genioplasty as an isolated surgical technique is a highly demanded procedure in the maxillofacial surgery area. Advances in facial reconstructive surgery have been associated with less morbidity and more predictable results. In this paper, “conventional” genioplasty and genioplasty by means of virtual surgical planning (VSP), CAD-CAM cutting guides, and patient custom-made plates are compared. Methods: A descriptive observational study was designed and implemented, and 43 patients were treated, differentiating two groups according to the technique: 18 patients were treated by conventional surgery, and 25 patients were treated through virtual surgical planning (VSP), CAD-CAM cutting guides, STL models, and titanium patient-specific plates. Results: The operation time ranged from 35 to 107 min. The mean operative time in the conventional group was 60.06 + 3.74 min.; in the custom treatment group it was 42.24 + 1.29 min (*p* < 0.001). The difference between planned and obtained chin changes in cases of advancement or retrusion was not statistically significant (*p* = 0.125; *p* = 0.216). In cases of chin rotation due to asymmetry, guided and personalized surgery was superior to conventional surgery (*p* < 0.01). The mean hospital stay was equal in both groups. A decrease in surgical complications was observed in the group undergoing VSP and customized treatment. Conclusions: Multi-stage implementation of VSP with CAD-CAM cutting guides, STL models, and patient-specific plates increased the accuracy of the genioplasty surgery, particularly in cases of chin asymmetry, reducing operation time and potential complications.

## 1. Introduction

Genioplasty consists of an osteotomy of the anteroinferior border of the mandible, allowing movement of the chin in the three dimensions of space and placing it in the desired new position. It is a highly in-demand procedure in the context of maxillofacial and plastic surgery. The technique was described in the 19th century, and it became popular in the last decades of the 20th century as a complement to the surgical correction of dentofacial deformities [1,2]. Progressively, surgical techniques have been refined from aggressive osteotomies with wire-type fixations through alloplastic grafts to chin osteotomy with semi-rigid osteosynthesis for the correction of chin projection and/or asymmetry. The increase in social demand for correcting dental and/or skeletal problems and the greater coordination between orthodontists and maxillofacial surgeons have led to the standardization of this procedure, associated or not, with mono- or bimaxillary orthognathic surgery within the usual care activity of oral and maxillofacial surgery departments [2,3,4,5].

Advances in reconstructive surgical techniques in the head and neck area have allowed a comprehensive treatment and a complete surgical approach, with significant improvement in aesthetic and functional reconstruction, fewer comorbidities, and better recovery for the patients [6]. Less aggressive surgical techniques, additive manufacturing, and digital workflow have significantly improved comprehensive reconstruction of the facial skeleton [5,6,7,8], including surgical treatment of the chin. In this context, the development of virtual surgical planning (VSP), CAD-CAM technology, and the manufacture of cutting guides and customized titanium mini-plates have brought about a paradigm shift in the surgical treatment of mandibular deformities. Semi-rigid fixation for chin osteotomy is considered the main osteosynthesis technique, although traditionally, the material for bone fixation was not customized [9]. This study compares two genioplasty techniques: conventional genioplasty by osteotomy and semi-rigid fixation with screws and mini-plates with “freehand” planning, and genioplasty by osteotomy with customized cutting guides and personalized plates prior to VSP.

## 2. Materials and Methods

To address the research purpose, the authors designed and implemented a descriptive observational study with 43 patients diagnosed with chin deformity (excess, defect, or asymmetry) between 2019 and 2023 at the Hospital Universitario Ruber Juan Bravo, Madrid, Spain. Eighteen patients were treated by means of the conventional genioplasty technique with osteotomy and semi-rigid fixation with non-customized material. The remaining 25 patients underwent surgery after virtual surgical planning (VSP) design and manufacture of specific cutting guides and titanium plates using CAD-CAM technology. Patients treated with conventional surgery were treated between 2019 and 2021.

Inclusion criteria were: (1) patients with a diagnosis of chin deformity; (2) patients reconstructed with the “conventional” technique; and (3) patients treated with VSP, custom cutting guides, and titanium plates. Exclusion criteria were: (1) patients with previous surgery or surgical sequelae affecting the mandibular region. Follow-up ranged from 6 to 50 months.

This study followed the Declaration of Helsinki on medical protocol, and the study and review of medical records, data collection, and subsequent analysis are endorsed by the Hospital Ethics Committee (protocol code rjbmaxilo 01/2024).

In the cases treated by traditional methods, a preoperative CT scan was performed on all patients in DICOM format, which was subsequently edited with open-access software in order to obtain a stereolithographic model (STL) with 3D printing in polyamide. A “model surgery” was performed on this template in order to propose the most suitable surgical movements in each case and thus decide on the optimal positioning of the chin. The titanium plates adapted to the patient’s anatomy were molded preoperatively. Once in the operating room, an intraoral approach was performed in all cases, identifying and preserving the chin nerves. The osteotomy line was marked, and the osteotomy was performed in the most appropriate region, taking into account the previous work on the 3D models. It was performed with a piezoelectric scalpel, and the fixation of the bone was made with mini-plates and titanium screws.

**Figure 1:**
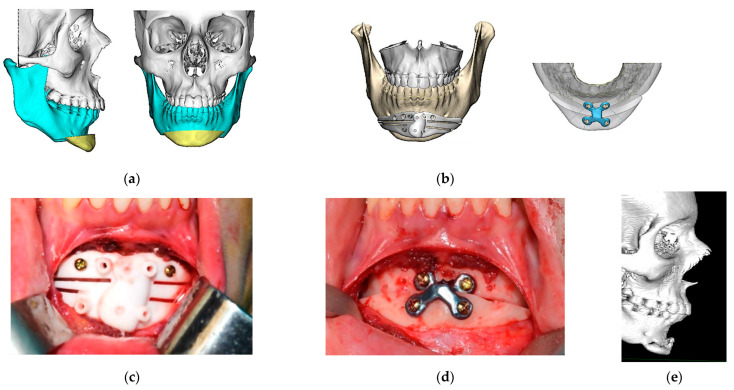
Customized guided surgery. (**a**) Virtual surgical planning and design of the osteotomy. Sagittal and front view; (**b**) digital design of cutting guide and titanium plate. Avinent Implant System S.L.U, Santpedor, Barcelona, Spain; (**c**) intraoperative photograph: fitting of cutting guide; (**d**) intraoperative photograph: final position of the chin with customized titanium plate and screws; (**e**) six-month post-operative control CT. Sagittal view.

In cases treated through VSP, cutting guides, and patient-customized plates, a remote connection was established with the engineering team of the corresponding manufacturer (Avinent Implant System S.L.U, Santpedor, Barcelona, Spain). The osteotomy cutting guides were designed using CAD-CAM technology and produced with a 3D printer in polyamide; a surface and volumetric measurement of the chin positioning was performed on the virtual model once the osteotomy had been delimited, and a customized titanium prosthesis was designed for each patient. The customized titanium plates were fabricated using CAD-CAM technology, and they were manufactured with a DMG HSC (high-speed cutting) milling machine (Avinent Implant System S.L.U, Santpedor, Barcelona, Spain). Irregular edges or protrusions that could compromise the patient’s comfort or aesthetic results were avoided. The optimal fit of the bone-cutting guides and the plates (Avinent Implant System S.L.U, Santpedor, Barcelona, Spain) was checked on printed STL models. An intraoral approach was performed in all cases. After identification and preservation of the mentonian nerves, the two-piece cutting guide was adapted, and the osteotomy was performed with a piezoelectric scalpel. The planned chin movement was performed, and semi-rigid osteosynthesis was effectuated with titanium plates and screws according to the manufacturer’s instructions (Figure 1).

All cases were also studied by preoperative and postoperative computed tomography (CT). Sagittal plane movement (advancement or retrusion) of the chin was measured in millimeters. In cases of asymmetry, chin rotation was measured in degrees, taking the nasion–chin vertical as a reference.

To generate the 3D anatomical model, the preoperative CT scan was obtained in DICOM format. The files were processed using the medical software Mimics Innovation© Suite 20.0. The virtual design of the sagittal movements of the chin was performed using Dolphin Imaging Software© (version 10.5, Canoga Park, CA, USA), modeling the target according to the patient’s wishes and the experience of the surgical team. Before fabrication of the cutting guides and custom mini-plates, the virtual design of all the material was checked by the surgeon before approval or modification.

The operative time was also measured in all cases. Moreover, all patients were asked about their perception of the aesthetic result 6 months after surgery.

The variables evaluated in this study were:

(1) Difference between planned and achieved motion with both techniques (measured in millimeters, and in degrees for chin rotation cases). The Phillips IntelliSpace Portal© V.11.1(Koninklijke Philips N.V. Amsterdam, The Nederlands). radiological viewer was used for this purpose.
(2) Intervention time and length of hospital stay between the two techniques.
(3) Aesthetic outcome: an aesthetic assessment was performed by the patients to address scores in facial symmetry, facial healing, and facial projection. The results were classified as “excellent”, “good”, and “poor”.

Statistical analysis was performed with SPSS© 28.0 software (IBM SPSS Statistics, Chicago, IL, USA).

## 3. Results

Forty-three patients were included in the study. Eighteen patients were treated by the conventional genioplasty technique, and 25 patients were treated with VSP, cutting guides, and a customized plate. Of the total sample, 40% (17 patients) were treated in the context of orthognathic surgery, while 60% of the patients underwent genioplasty for strictly aesthetic reasons.

Only 14% were men (6 patients). The mean age of the patients in the “conventional surgery” group was 28.89 ± 2.26 years; the mean age of the “guided surgery” group was 31.44 ± 1.76 years.

To simplify the study, patients were divided into subgroups: advancement (19 patients), retrusion (13 patients), and rotation (11 patients). The advancement, retrusion, or twist motion is shown in Table S1 (customized guided surgery) and Table S2 (conventional surgery).

### 3.1. Differences between Planned and Achieved Surgical Motion

The differences between planned and achieved surgical motion (in millimeters) by group is shown in Table 1. There were no significant differences in the comparison of the two techniques with respect to chin advancement or retrusion. However, statistical significance was observed in cases of mandibular asymmetry that required chin rotation (*p* = 0.011).

### 3.2. Surgical Time and Hospitalization

**Figure 2:**
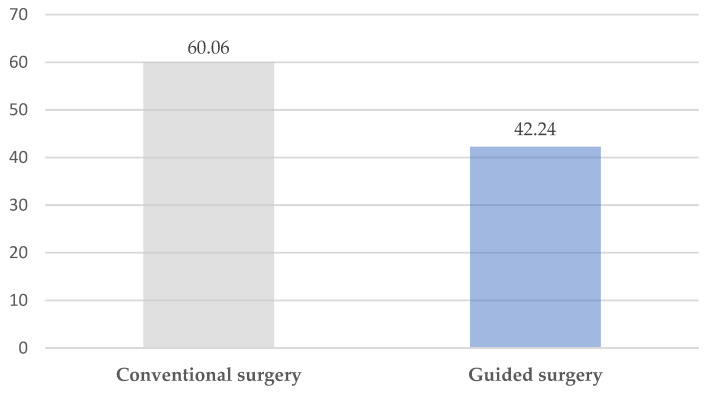
Surgical time (minutes).

The operation time ranged from 35 to 107 min. The mean operative time was 60.06 ± 3.74 min in the conventional surgery group and 42.24 ± 1.29 min in the group treated by VSP, CAD-CAM cutting guides, and a customized plate. There was a considerable decrease in surgical time in the cases treated by guided surgery and individual plate (*p* < 0.001) (Figure 2).

The mean hospital stay was 1.4 days, with no differences between groups (*p* = 0.196).

### 3.3. Aesthetic Outcome: Patient Perception

Eighty-eight percent of the patients (38 cases) considered that they had obtained an “excellent” aesthetic result. Twelve percent (5 cases) evaluated their result as “good”. There was no difference between the group treated with conventional surgery and the group treated with customized guided surgery (*p* = 0.22). There was also no statistical difference by subgroup (Table 2).

## 4. Discussion

The development and refinement of techniques and methods due to technological advances have allowed surgical procedures to be performed with less morbidity, more accurately, and more safely. The use of computer-assisted surgery and navigation technology in head and neck surgery was described at the end of the 20th century by A. Wagner. In recent years, “precision medicine” has been integrated into daily hospital medical practice, allowing the development of specific and customized materials for each case. In this context, maxillofacial surgery as a discipline has benefited from this technological development: virtual surgical planning and the design and manufacture of customized CAD-CAM material have contributed, in recent years, to simplifying and improving the precision of surgeries. Together, all this makes it possible to virtually plan the limits of an osteotomy or a resection, to know the dimensions of a potential defect, to determine the precise location of osteotomies in bone flaps, etc. Cutting guides manufactured using CAD-CAM technology help the surgeon faithfully reproduce the previously planned treatment, improving the precision, accuracy, and reliability of the results in resections and reconstructions of the head and neck area. The combination of VSP and CAD-CAM technology can provide the best possible postoperative results, especially in complex cases without adequate intraoperative anatomical references [10].

There are not many studies describing the use of customized plates and cutting guides in the context of genioplasty; however, there has been an increase in interest in this area in recent years that will make the technique, still susceptible to improvement, be enhanced in the near future. The growing interest and progressive application of virtual surgical planning and the use of customized titanium mini-plates manufactured using CAD-CAM technology have developed in recent years in orthognatic surgery [11,12,13].

The systematization of the process described in our patients has allowed us to ensure a better result, to increase the precision and safety of the surgery, and to reduce operating room time. This leads to avoid potential peri- and postoperative complications.

**Figure 3:**
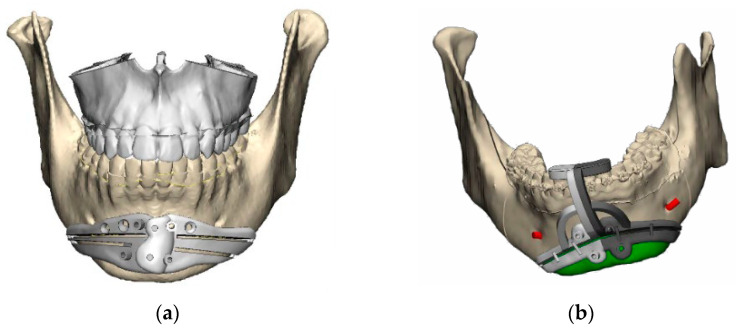
Customized guides (Avinent Implant System S.L.U, Santpedor, Barcelona, Spain): (**a**) polyamide customized cutting guide with mandibular adjustment; (**b**) polyamide customized cutting guide with mandibular and teeth adjustment.

Adequate design of the cutting guide is crucial to obtaining a good result. Although it is important to correctly delimit the placement of the osteotomy, it is equally important to take into account the ergonomics and adaptation of the guide to the patient’s jaw so as not to collaterally damage important anatomical structures [14]. In our experience and taking into account the idiosyncrasies of each patient as well as the surgeon’s preferences, we have designed two types of polyamide cutting guide. One type consists of a two-piece system with intermediate connections and adaptations to the mandibular basal that allow its introduction through a relatively small surgical wound. The other type also has an extension with adjustments to the incisal edge of the teeth of the lower arch to increase stability (Figure 3). In addition, in the design of the guide, it is of vital importance to distribute the holes for fixation to the bone in positions that do not compromise the aesthetic result or damage the mentonian nerves or the floor of the mouth due to excessive length. Attention should also be paid to the length of the titanium screws that will be used to fix the custom osteosynthesis plate. In general, the mandibular bone has a high density, and the bone volume at the mental level is large. However, if the anatomy of each case is not taken into account, it is possible to place screws that are longer than the bone width and cause injury to soft structures, with a potential risk of bleeding, especially the floor of the mouth and the mental area [15,16].

The use of polyamide as a material for the manufacture of the customized cutting guides also provides a small modulus of elasticity that enhances the positioning of the instrument at the surgical site and its subsequent adaptation [17,18]. This also makes it possible to maintain a small approach and an earlier recovery of the surgical wound.

We believe there are many advantages to using customized cutting guides when performing mandibular osteotomy. In the conventional technique, the design of the osteotomy is performed “free-hand” according to the surgeon’s preference and previous experience; however, the cutting guide ensures an osteotomy path that always avoids the mentonian nerves and the dental apices since they have been previously analyzed in 3D. In addition, it ensures the symmetry of the osteotomy and the precision of the midline positioning. Therefore, the centering of the mentonian bone fragment will also be better positioned [19].

Of course, in cases of asymmetric, complex chins, the placement of the fragment will be much simpler using customized material for the patient according to a previous VSP and will generate less doubt among the surgical team about the final result. This considerably reduces surgical time, as we have demonstrated, and by avoiding excessive manipulation of the soft tissues in cases of disagreement with a conventional procedure, postoperative edema and the sequelae derived from it (pain, swelling, patient discomfort, etc.) are reduced [20]. In our series of cases, there were no statistically significant differences in the cases of chin advancement or retrusion. In this regard, we consider that this may be due to the fact that the movement achieved is equally precise with a previously calibrated chin advancement or retrusion plate. In fact, although it has no clinical relevance, these results may induce the thought that surgery has no benefit in some cases. We believe that the results in simple advancements and retrusions may be slightly inferior in terms of radiologic measurement and comparison—not clinical—than the results obtained in the guided procedures because the latter ones had higher complexity. However, significant differences were found in cases of asymmetry requiring chin rotation, which confirms that planning is especially useful for cases in which the movement is not only in the sagittal plane but in all three dimensions of space.

Undoubtedly, developing genioplasty surgery with cutting guides and customized titanium plates decreases miscalculations and complications with respect to the traditional technique. In the latter case the osteotomy is designed and executed freehand and the fragment is positioned according to the approximate measurement made during the procedure, without having a complete visualization of the structure, which can lead to asymmetric and/or unexpected results, often unacceptable for the patient and the surgeon [21,22].

Complications of genioplasty due to damage to the mentonian nerve or tooth apices are described in the scientific literature with a variation of 3% to 12% [14,23,24]. Although we consider that these are not frequent complications and that, with wide experience, they can be avoided, the safety and precision provided by VSP and the use of this technology during surgical manipulation are unequaled.

In our series, no differences were found with respect to complications, which fortunately were few, although we consider that the safety that the use of customized cutting guides brings to the procedure results in a clear reduction of potential morbidity for the patient. The only relevant complication was a case of mild submental hematoma that was solved conservatively in the conventional surgery group. There were no complications in the group treated with VSP and cutting guides.

In this sense, the aesthetic results obtained are equal to or better than those of the conventional technique and are always more predictable [7,14]. The reduction in surgical time and tissue manipulation resulted in a better and shorter recovery for the patients, although this was a subjective finding on the part of both the patients and the medical team. In any case, the patients’ perception of having undergone a more pleasant postoperative period with less swelling than they had expected made their postoperative management as well as their reincorporation into daily life easier.

Related to the use of customized cutting guides, we can affirm that they allow us to design more complex osteotomy paths and to “take better advantage” of the patient’s mandibular bone, being able to design more posterior cutting paths that provide more volume and thus avoid the “hourglass” deformity typical of some cases with poor planning [21,24,25].

In addition, in cases of chin advancement and retrusion, the posterior wedges, which are sometimes produced by the movement of the cut area of the bone, are included in the design of the cutting guide, making it possible to achieve a more natural mandibular ridge with a higher-quality aesthetic result. At this point, we consider it important to explain to each patient the steps of their surgical treatment and the normal postoperative findings. In many patients with excellent functional and aesthetic results, there is a complaint of a bony step in the bilateral basal area of the osteotomy. Planned surgery and the use of customized cutting guides allow, in most cases, the design of more posterior osteotomies with a less pronounced gradient toward the mandibular basal that will produce more discrete gaps. However, we insist on informing patients of this possible palpable transition prior to surgery.

In cases of chin asymmetry and also those of mandibular profile, the design of customized cutting guides allows the mandibular contour to be modified with great precision and safety; this would not be possible without the guide since the mandibular basal cannot be visualized during the procedure.

On the other hand, the use of customized titanium plates brings advantages. It reduces the surgical time because, as mentioned above, the placement of the mentonian bone fragment is faster and more precise, and, above all, it will have a positioning in the three dimensions of the space previously defined. In addition, the passive adaptation to the bone surface of the chin and jaw allows for smoother osteosynthesis without potential changes of the designed chin position due to forceful movements to achieve a tighter fit of the titanium screws, thus accidentally bending the titanium mini-plates previously molded on the 3D model or on the patient’s bone [10,18].

In our series of cases, a considerable reduction in surgical time has been demonstrated, with the advantages that this entails for the patient. There was no difference in hospitalization time between one case and another. We relate this result to the fact that with both techniques, it is a less aggressive procedure. In addition, many patients who were admitted to the hospital for 1 or 2 days did so for personal reasons and not strictly medical reasons.

The use of VSP, CAD-CAM technology, and customized material allows us to achieve an adequate aesthetic result of the mandibular contour in cases in which the traditional technique produces an unsatisfactory or unpredictable result [21]. It is important to emphasize the high aesthetic demand presented by patients who wish to undergo this procedure in a stand-alone fashion and for aesthetic reasons only.

Despite the great advantages of using this technology, some disadvantages and limitations should also be clarified. First, more time is required for the design of the VSP and the additive manufacturing of the customized models, guides, and plates. We agree with other authors that the whole process can be solved within 48 h [14,26,27]. However, we believe the surgical time saved compensates for the planning effort. This waiting time until virtual surgical planning and obtaining custom cutting guides and titanium plates can be an inconvenience in other contexts. We must consider that this technology applied to the field of genioplasty can be extended to other surgeries in the head and neck area. In interventions that need to be carried out as early as possible (emergencies, oncology patients with potential complex reconstructions of the facial skeleton, accidents in the context of the postoperative period of a surgery that has previously required customized material, etc.), there is the possibility of waiting for the design of the material or, on the contrary, it may be more appropriate to carry out a conventional treatment.

It should be taken into account that the design will not allow intraoperative modifications in case of any error, so that a failure in the planning or an error in the production of the material will mean the need to change the entire preoperative plan. The customized plate must also fit perfectly passively, since it will not allow intraoperative modifications. In these cases, it will be necessary to use one or more conventional osteosynthesis plates [14,28,29].

Although there is an additional cost with the use of this technique (400–1000 euros, according to the authors), there is a savings in anesthetic support in cases performed under local anesthesia and sedation [26,30,31]. In cases performed under general anesthesia, the savings in surgical time will not compensate for the economic expense, but there is a benefit in terms of patient safety and results.

We should mention the limitations of this study. Firstly, this is a study with a small number of cases, so the scope of the results obtained is small. On the other hand, the constant refinement and improvement of the methods means that in each case, details are incorporated into the design of the cutting guides and/or the customized titanium plates that may surpass the previous ones. Although the results obtained are highly satisfactory at the present time, it is expected that a gradual improvement will allow, in a few years, the definition of a standard protocol for guided genioplasty surgery.

Currently, we consider this genioplasty technique superior to the conventional one, so the cases included in this work that were treated by conventional technique are prior to the rest.

## 5. Conclusions

The multistage application of VSP with CAD-CAM polyamide cutting guides, STL models, and patient-specific titanium plates increases precision in genioplasty surgery, especially in cases of chin asymmetry, reducing operating time and possible complications. The main advantages of this technology are the visualization and preoperative study of the anatomy of each patient, the osteotomy with precise and well-defined limits, the simplification of the surgical technique, the preoperative visualization of the limitations of the case and prevention of potential complications, the reduction of surgical time, and the obtaining of more predictable results. On the other hand, the main disadvantages of virtual surgical planning are increased costs and surgical delays involved in surgical planning and obtaining the different models and guides. Overall, the decrease in patient morbidity and complications and the overall improvement in outcomes could offset the technological costs. Further studies with larger series of patients are needed, as well as improved techniques to standardize the guided surgery protocol to provide better outcomes.

## Figures and Tables

**Table 1:** Differences between planned and achieved surgical motion.

	Conventional Genioplasty	Guided Genioplasty	*p* Value
Advance (mm.)	0.13 ± 0.05	0.46 ± 0.02	0.125
Retrusion (mm.)	0.18 ± 0.5	0.10 ±0.06	0.216
Midline change (degrees)	2.60 ± 0.51	0.50 ± 0.22	0.011

**Table 2:** Aesthetic results: patient perception.

	Excellent	Good	Poor
Conventional genioplasty	15	3	0
Guided genioplasty	23	2	0

## Data Availability

The data presented in this study are available on request from the corresponding author. The data are not publicly available due to data protections regulations.

## Supplementary Materials

The following supporting information can be downloaded at: https://www.mdpi.com/article/10.3390/jpm13121702/s1. Table S1: Patients treated by customized guided surgery; Table S2: Patients treated by conventional surgery.
