# Supplementary material for: Guided Genioplasty: Comparison between Conventional Technique and Customized Guided Surgery
Source: J Pers Med. 2023 Dec 12;13(12):1702. doi: 10.3390/jpm13121702 (PMC10744815; doi:10.3390/jpm13121702)
Supplement: Supplementary file 1 [file jpm-13-01702-s001.zip › jpm-2734309-supplementary.pdf]

Table S1. Patients treated by customized guided surgery.

Guided surgery	Age (y)	Sex	Advance pre. (mm.)	Retrusion pre. (mm.)	Midline change pre. (mm.)	Midline change post. (mm.)	Surgical time (min.)
Patient 1	34	Woman	5			5.1	38
Patient 2	35	Man	5			5	34
Patient 3	18	Woman	7			6.8	43
Patient 4	24	Woman	7			6.9	46
Patient 5	22	Man	3			3	55
Patient 6	48	Woman	5			5	40
Patient 7	20	Woman	4			4	39
Patient 8	23	Woman	5			5.2	40
Patient 9	27	Man	7			7.1	51
Patient 10	27	Woman	5			5.2	35
Patient 11	30	Woman	5			5	27
Patient 12	25	Man	7			7	41
Patient 13	40	Woman	5			5	36
Patient 14	41	Woman			15º	16º	48
Patient 15	37	Woman			14º	14º	51
Patient 16	21	Woman			10º	11º	45
Patient 17	25	Woman			16º	16º	46
Patient 18	29	Woman			20º	21º	42
Patient 19	40	Woman			18º	18º	48
Patient 20	51	Woman		3		3.4	42
Patient 21	28	Woman		4		4	50
Patient 22	39	Woman		3		3.1	46
Patient 23	37	Woman		5		5.1	35
Patient 24	28	Woman		5		5	40
Patient 25	37	Woman		3		3	38

Table S2. Patients treated by conventional surgery.

Conventional surgery	Age (y)	Sex	Advance pre. (mm.)	Retrusion pre. (mm.)	Midline change pre. (mm.)	Midline change post. (mm.)	Surgical time (min.)
Patient 1	19	Woman	5			5.2	46
Patient 2	34	Man	7			6.8	45
Patient 3	19	Woman	6			6.2	47
Patient 4	28	Woman	3			3.2	58
Patient 5	38	Woman	5			5.3	62
Patient 6	35	Woman	5			5	69
Patient 7	30	Woman	6			5.8	57
Patient 8	21	Woman		3		3.1	49
Patient 9	49	Man		5		5.2	55
Patient 10	45	Woman		4		4.3	54
Patient 11	39	Woman		5		4	55
Patient 12	37	Woman		3		3	67
Patient 13	23	Woman		5		5.2	54
Patient 14	20	Woman			15º	17º	87
Patient 15	18	Woman			14º	18º	61
Patient 16	21	Woman			22º	25º	107
Patient 17	22	Woman			21º	22º	45
Patient 18	24	Woman			19º	22º	68
